# Late-Life Drinking Behavior: The Influence of Personal Characteristics, Life Context, and Treatment

**Published:** 1996

**Authors:** Penny L. Brennan, Rudolf H. Moos

**Affiliations:** Penny L. Brennan, Ph.D., is a health science specialist at the Program Evaluation and Resource Center and the Health Services Research and Development (HSR&D) Center for Health Care Evaluation, Palo Alto VA Health Care System and Stanford University Medical Center, Palo Alto, California. Rudolf H. Moos, Ph.D., is director of the Program Evaluation and Resource Center and the HSR&D Center for Health Care Evaluation, Palo Alto VA Health Care System and Stanford University Medical Center, Palo Alto, California

**Keywords:** elderly, problematic AOD use, demographic characteristics, environmental factors, life event, treatment, psychological stress, social support, comorbidity, coping skills, AOD consumption, AODU development, predictive factor, health care utilization, longitudinal study, scientific model

## Abstract

Alcohol-related problems affect increasing numbers of older adults. Recent studies have begun to investigate problem drinking among older adults based on a conceptual model proposing correlations between personal characteristics, life context (i.e., environmental factors), treatment, and drinking-related outcomes. In a community sample of older problem and nonproblem drinkers, alcohol consumption, life stressors, social resources, and coping responses differed between the two groups, although these factors did not directly and uniformly affect late-life drinking behavior. Furthermore, drinking behavior did not always have the expected effects on older drinkers’ life contexts. Findings from a sample of treated alcohol and other drug-abusing older patients demonstrated the importance of providing mental health aftercare for this group.

Historically, most efforts to understand and treat problem drinking^1^ have focused on young and middle-aged adults. However, two factors have prompted increased attention to the alcohol-related problems of older adults.^2^ First, clinicians show a growing concern over the prevalence of these problems among their older patients. Second, researchers anticipate increasing prevalence of late-life problem drinking because of the currently large number of middle-aged adults and their present levels of alcohol use (Adams and Cox 1995; Beresford 1995).

This heightened interest in the alcohol-related problems of older adults has inspired studies yielding considerable information about the scope and correlates of alcohol consumption and problem drinking in late adulthood. The current prevalence of alcohol abuse and dependence in the general population of older adults is between 2 and 4 percent; it is considerably higher—5 to 30 percent—in clinical populations (American Medical Association [AMA] Council on Scientific Affairs 1996; Adams and Cox 1995). Most studies have found that older adults consume less alcohol and have fewer drinking problems than do younger adults. Research has repeatedly shown that alcohol consumption and drinking problems are greater in older men than in older women. The literature also shows that late-life problem drinking is associated with a host of cognitive and health problems as well as with an increased risk of adverse medication-alcohol interactions (for reviews, see Adams 1995; AMA Council on Scientific Affairs 1996; Bucholz et al. 1995; Smith 1995; Tartar 1995).

Recent research highlights the importance of distinguishing between two groups of older problem drinkers: late onset and early onset. Late onset problem drinkers, of whom a significant proportion are women, first develop drinking problems later in life (i.e., after age 50). Early onset problem drinkers, in contrast, develop drinking problems earlier during adolescence or adulthood and maintain them into late life. Compared with early onset problem drinkers, late onset problem drinkers generally are in better health (e.g., have fewer physical symptoms and experience less depression), have better social relations (e.g., are more likely to be married and less likely to be incarcerated), and are less likely to have been treated for alcohol and other drug (AOD) abuse (for reviews, see Atkinson 1995; Liberto and Oslin 1995; Schonfeld and Dupree 1991). Late onset problem drinkers, who constitute one-third to one-half of older problem drinkers, also may have a better prognosis, possibly because interventions are more effective before severe alcohol problems have robbed the drinkers of their physical and social resources (Liberto and Oslin 1995).

Despite the progress in this research area, many gaps remain in the knowledge about late-life problem drinking. For example, most researchers have conducted their studies at a single point in time (i.e., have performed cross-sectional analyses), preventing inferences about the direction of causality between variables (e.g., do personal characteristics influence drinking problems, or do drinking problems influence personal characteristics?). Moreover, most studies have focused on correlations between personal characteristics (e.g., age, gender, and time-of-onset of drinking problems) and late-life drinking behavior; few have considered the influence of the drinkers’ environmental contexts (e.g., life stressors and social resources). Finally, little is known about either the factors that prompt older adults to seek help for their alcohol-related problems or the treatment efficacy for this group. This article presents a model for conceptualizing late-life drinking behavior. Based on this research model, the article also describes the findings of two long-term (i.e., longitudinal) research studies aimed at elucidating predictors of alcohol consumption, drinking problems, treatment seeking, and treatment outcome in older adults.

## A Model of Late-Life Drinking Behavior

**Figure 1:**
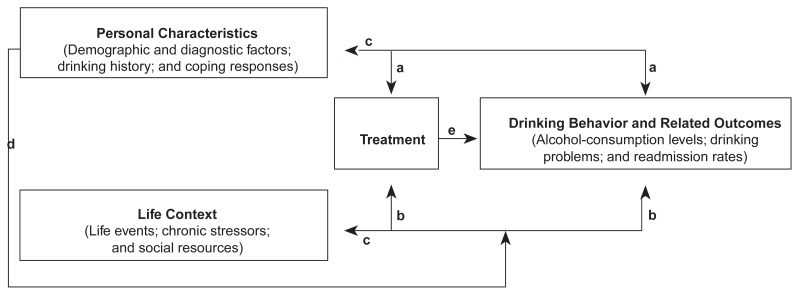
A conceptual model of late-life drinking behavior depicting the relationships between personal characteristics, life context, treatment, and drinking behavior and related outcomes. Path a represents the influences of personal characteristics on, and path b denotes the influences of life context on, treatment and drinking behavior. Path c indicates that drinking behavior can affect personal characteristics and life context. Path d represents the moderating effects of personal characteristics on the relationship between life context and drinking behavior. Path e signifies the connection between treatment and drinking-related outcomes.

The conceptual framework presented in figure 1 suggests that three major sets of factors affect late-life drinking behavior and related outcomes: personal characteristics, life context, and treatment. Personal characteristics that can influence drinking behavior include a person’s demographic attributes (e.g., gender and ethnicity), diagnosis (e.g., coexisting psychiatric conditions or depressive symptoms), history of drinking behavior (e.g., volume of alcohol consumption and chronicity of drinking problems), and coping strategies used to manage stressors. The term “life context” refers to a person’s environment; the concept includes stressors (i.e., acute negative life events and chronic stressors) and social resources (e.g., perceived emotional support) in several life domains, such as health, finances, home and neighborhood, work, and interpersonal relationships. The life context also includes significant others’ use of and attitudes about alcohol (e.g., friends’ approval of drinking). The treatment category considers all previous treatment experiences for AOD-abuse problems, including treatment seeking and specific treatment program characteristics. Together, these three sets of factors shape a person’s drinking behavior and related outcomes, including alcohol consumption, drinking problems, and hospital readmission.

The late-life drinking behavior model posits several relationships between these four domains (i.e., personal characteristics, life context, treatment seeking, and drinking behavior):

- Both personal characteristics and life context affect treatment seeking as well as drinking behavior and outcomes.
- Drinking behavior, in turn, influences a person’s life context and certain personal characteristics.
- Some personal characteristics moderate the relationship between life context and outcomes.
- Treatment, including specific aspects of treatment programs, influences drinking-related outcomes.

The validity of these assumptions was tested using a sample of older community residents as well as a sample of treated older AOD-abuse patients. The findings obtained with these two samples are described in the following sections.

## Relationships Between Personal Characteristics, Life Context, Treatment Seeking, and Drinking Behavior Among Community Residents

To examine the relationships between personal characteristics, life context, treatment seeking, and late-life drinking behavior, Brennan, Moos, and colleagues (Brennan and Moos 1990; Moos et al. 1990) studied a sample of 1,884 older community residents (i.e., people 55 to 65 years old when the study began). The participants completed extensive surveys concerning life stressors, social resources, coping responses, drinking behavior, and health at the beginning of the study as well as 1 and 4 years later. At the initial assessment, the respondents were classified into three drinking categories: (1) remitted problem drinkers^3^ (i.e., individuals who had had drinking problems in the past but had no current problems), (2) problem drinkers (i.e., participants with one or more current drinking problems), and (3) nonproblem drinkers (i.e., people with no past or current drinking problems). (For details about the sampling and group classification procedures, see Brennan and Moos 1990; Moos et al. 1990.)

Focusing on the latter two groups, the investigators addressed four questions: (1) Do the personal characteristics, life contexts, and coping responses of older problem drinkers differ from those of older nonproblem drinkers? (2) Do personal and life context factors at the beginning of the study predict drinking behavior 1 and 4 years later? (3) How does initial alcohol use in older problem drinkers affect their subsequent life context and psychological well-being? (4) What factors prompt older community residents to seek treatment for alcohol-related problems?

### Differences Between Older Problem and Nonproblem Drinkers

Older problem and nonproblem drinkers differed with respect to several personal characteristics (Brennan and Moos 1990). As expected based on previous studies, more men than women were classified as problem drinkers. Problem drinkers were more likely to be unmarried than nonproblem drinkers; they also consumed twice as much alcohol as nonproblem drinkers did. Finally, nonproblem drinkers by definition reported no negative consequences of their drinking behavior, whereas problem-drinking men reported an average of 5.5 and problem-drinking women reported an average of 4.2 current drinking-related problems.

**Figure 2:**
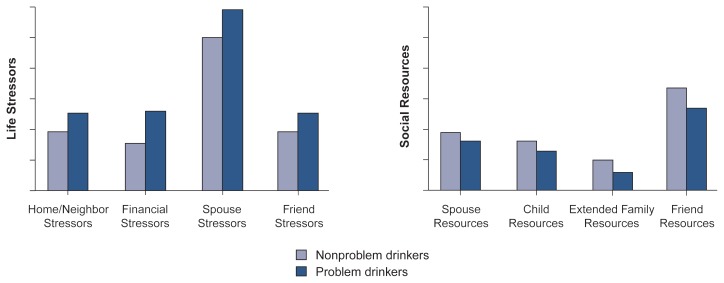
The relative levels of life stressors and social resources of problem and nonproblem drinkers in a community sample of older adults. SOURCE: Brennan and Moos 1990.

Problem drinkers also had more stressful life contexts than did nonproblem drinkers (figure 2). Thus, they experienced more chronic stressors, such as ongoing adversity involving home and neighborhood (e.g., lack of quiet and safety), financial problems, and persistent interpersonal conflicts with spouses and friends. Furthermore, problem drinkers had fewer social resources. For example, they reported less support from their spouses, children, extended family, and friends than did nonproblem drinkers (Brennan and Moos 1990).

**Figure 3:**
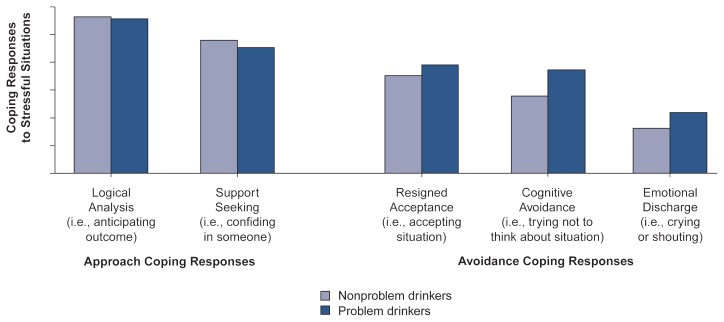
Coping responses of nonproblem and problem drinkers in a community sample of older adults. Both groups frequently used approach coping responses, such as logical analysis and support seeking. However, problem drinkers were more likely than nonproblem drinkers to use avoidance coping responses, such as resigned acceptance, cognitive avoidance, and emotional discharge. SOURCE: Moos et al. 1990.

Finally, problem drinkers and nonproblem drinkers differed in their coping responses to stressful situations. Two broad categories of coping responses are approach coping and avoidance coping. Approach coping involves attempts to master or resolve a stressful situation; avoidance coping consists of efforts to avoid thinking about a stressor and its implications and includes the expression of stress-related emotions without attempting a resolution. A comparison of older problem and nonproblem drinkers showed that although both groups used comparable levels of approach-coping strategies to manage stressors, problem drinkers were more likely to use avoidance-coping strategies (figure 3) (Moos et al. 1990).

Consistent with earlier research (Atkinson 1995; Liberto and Oslin 1995; Schonfeld and Dupree 1991) several differences existed between the late onset and early onset problem drinkers in the sample (Brennan and Moos 1991). Compared with early onset problem drinkers, late onset problem drinkers consumed less alcohol, had fewer drinking problems, reported fewer physical symptoms and stressors involving friends, and received more emotional support from children and friends.

Overall, these results implied that experiencing more stressors, having fewer social resources, and relying more heavily on avoidance coping could lead to late-life drinking problems. The results also suggested that drinking problems might harm the life contexts and psychological well-being of older adults. To learn more about these causal relationships, the participants were reassessed over extended periods of time.

### Predictors of Alcohol Consumption and Drinking Problems

#### Stressors

The “stress hypothesis” suggests that increased alcohol consumption and drinking problems in older adults are direct responses to heightened environmental stressors, such as the deaths of significant others and increased health and financial problems (for a review, see Osgood et al. 1995). Empirical research findings indicate, however, that the relationship between stressors and late-life drinking behavior is more complex than the stress hypothesis suggests. For example, several longitudinal studies show that health-related stressors predict *reduced* alcohol consumption over intervals ranging from 1 to 10 years (Glass et al. 1995; Hermos et al. 1984). Other cross-sectional studies (e.g., Kasl et al. 1987; LaGreca et al. 1988; Welte and Mirand 1995), as well as longitudinal analyses of the community sample (Brennan et al. 1994; Brennan and Moos 1996), have detected no link between the number of non-health-related negative life events and subsequent alcohol consumption. Thus, health stressors may suppress older adults’ alcohol consumption, whereas non-health-related stressors alone (i.e., in the absence of other risk factors) appear to have little effect on older adults’ alcohol consumption.

In contrast to alcohol consumption, drinking problems appear to be more readily affected by life stressors. For example, among older adults in the community sample, a larger number of negative health events at the beginning of the study predicted fewer drinking problems 1 and 4 years later (Moos et al. 1991; Schutte et al. 1994). Other types of stressors, however, appear to elicit increased drinking problems in later life. For example, in the longitudinal Normative Aging Study, retirees were three times more likely to report the development of new alcohol problems than were people who had not retired (Eckert et al. 1989). Similarly, among older problem drinkers, non-health-related negative life events, as well as heightened friend and spouse stressors, presaged increased drinking problems at followup (Brennan et al. 1994; Brennan and Moos 1996).

#### Social Resources and Drinking History

If stressors can elicit late-life problem drinking, one might suppose that social resources can protect against it. Longitudinal analysis of the community sample of older adults showed, however, that a person’s drinking history (i.e., early onset versus late onset problem drinking) tends to determine how social resources influence drinking behavior (Moos et al. 1991; Schutte et al. 1994). Thus, initial social resources, such as emotional support from spouses and friends, did not predict drinking behavior 1 and 4 years later in early onset problem drinkers. In contrast, late onset problem drinkers who initially reported fewer social or financial resources were significantly more likely to abstain or to be in remission^4^ at 1- or 4-year followup than were late onset drinkers with greater initial resources. These findings suggest that among late onset problem drinkers, lost or reduced social support improves drinking behavior, whereas older adults who are early onset problem drinkers are less responsive to social resources.

The study’s findings also illustrate the influence of an older person’s drinking history on the relationship between life context and drinking behavior: The more alcohol the older drinkers consumed at the beginning of the study, the more likely were certain stressful life events, such as a relative’s sickness or injury, to cause an increase or a smaller-than-expected decrease in alcohol consumption at followup (Glass et al. 1995). Similarly, Brennan and colleagues (1994) found that although lighter drinkers tended to curtail their alcohol consumption in response to more acute health stressors, heavier drinkers did not.

#### Coping Responses

The use of certain coping responses also appears to moderate the effects of life context on late-life drinking behavior. For instance, more non-health-related negative life events predicted elevated drinking problems among older drinkers who relied heavily on avoidance coping. In contrast, among drinkers who used fewer avoidance-coping strategies, negative life events prompted a decline in drinking problems (Brennan and Moos 1996). Similarly, the influence of friends’ approval of drinking on the alcohol consumption of older problem drinkers depended on the extent to which the drinkers relied on avoidance coping to manage life stressors (Brennan et al. 1994).

Taken together, these observations belie the idea that life context directly and uniformly affects drinking behavior in later life. Whether and how stressors and social resources influence late-life drinking appear to depend on several factors, including (1) the specific type of stressors (i.e., health versus interpersonal stressors), (2) the type of drinking behavior being assessed (i.e., alcohol consumption versus drinking problems), and (3) personal risk factors (e.g., a longer history of problem drinking or use of avoidance-coping responses).

### Effect of Drinking Behavior on Life Context

Although several studies have analyzed the effects of life context on late-life drinking, few have considered the effects of older adults’ drinking behavior on their subsequent life contexts and personal well-being. Contrary to intuitive expectations, these studies indicate that ongoing drinking problems generally do not adversely affect the life contexts of older drinkers. For example, at the 1-year followup in the community study of older adults, problem-drinking women experienced a decline in spouse stressors, and problem-drinking men reported a reduction in conflicts with friends (Brennan et al. 1993). Moreover, the social resources of women with ongoing drinking problems remained relatively stable over the 1-year interval, although men with ongoing drinking problems lost support from their children. Thus, in the short term, older problem drinkers’ alcohol use may reduce interpersonal conflict and facilitate family functioning (Steinglass et al. 1987).

Similarly, older adults’ drinking behavior affected their psychological well-being in unexpected ways. For example, heavier initial alcohol consumption among women predicted fewer subsequent depressive symptoms; among men, more initial drinking problems predicted reduced depression (Schutte et al. 1995).

Finally, one would expect remission from drinking problems to improve the life contexts of older men and women. However, remission had little influence on the life contexts of male problem drinkers. Moreover, women who remitted experienced a loss of support from extended family members and reported more family stressors at followup than did remitted men (Brennan et al. 1993). Thus, for women, remission may entail costly changes in family context.

Longer term followups are needed to determine the permanence of these effects as well as their consequences. For example, do ongoing drinking problems eventually lead to increased interpersonal stressors? And do adverse family contexts or self-medication with alcohol to avoid depression pose relapse risks for older, remitted women? The answers to these questions might have treatment implications; older women in early remission, for example, may need enhanced support to cope with family conflict and depressive symptoms.

### Predictors of Treatment Seeking

Older adults rarely seek formal treatment (George et al. 1988). Consistent with this tendency, few problem drinkers in the community sample of older adults sought help for their alcohol-related problems. Thus, at initial assessment only about 4 percent of the late onset and 12 percent of the early onset problem drinkers had sought help in the past year specifically for drinking problems. Approximately one-fourth of these drinkers, however, reported seeking help for personal or emotional problems from a mental health professional or spiritual advisor (Brennan and Moos 1991).

Several factors predicted whether these community residents sought treatment. The most important predictors were prior treatment seeking and a greater number of health problems compared with the rest of the group. Frequently using avoidance-coping responses, experiencing more negative life events, and having fewer friends who approved of drinking also predicted more treatment seeking (Brennan et al. 1994). Finally, heightened spouse and health stressors at baseline foreshadowed more treatment seeking 4 years later (Brennan and Moos 1996).

These findings suggest that older adults in the community are reluctant to identify themselves as having drinking problems and therefore delay treatment until their health problems and stressful life contexts require it. This result highlights the importance of identifying and treating older adults’ drinking problems early. In addition, more information is needed about the personal characteristics, life contexts, and treatment features that deter older adults from seeking help for alcohol-related problems.

## Predictors of Treatment Outcome Among the Patient Sample

Despite their reluctance to seek treatment, older adults constitute at least 20 percent of the AOD-abuse population in inpatient settings (Kiesler et al. 1991). Yet little is known about these patients and their treatment outcomes. To investigate these issues, Moos and colleagues (1993) conducted a longitudinal study of 22,678 older (i.e., age 55 and over) AOD-abuse patients treated at Department of Veterans Affairs (VA) medical facilities. Most of the participants were male (99 percent), Caucasian (80 percent), and unmarried (60 percent); almost all of the participants had alcohol-related diagnoses. Both diagnostic and treatment information about these patients were obtained from VA computerized inpatient and outpatient databases for a period of 4 years before and 4 years after an index episode of inpatient care (i.e., the first episode of AOD-abuse-related treatment during the fiscal year 1987). (For further details, see Moos et al. 1993). For a separate group of 5,621 patients, detailed information was obtained about the treatment programs in which they participated (Moos et al. 1995).

Based on this information, the researchers addressed two broad sets of questions: (1) What are the extent and the predictors of health service use by older AOD-abuse patients? (2) To what extent is AOD-abuse treatment effective for older patients, and which specific treatment program characteristics predict better outcome?

### Extent and Predictors of Health Service Use

The VA AOD-abuse patients used health care services heavily. They received more than 920,000 days of care for AOD abuse or psychiatric disorders in the 4 years before their index episodes of care as well as 1.2 million days of care in the 4 years afterwards (Moos et al. 1994*b*). Moreover, readmission rates indicated that the treatment effectiveness was relatively low. Thus, 37 percent of the patients with only an AOD-abuse diagnosis (i.e., without other psychiatric disorders) at baseline were readmitted within 1 year, and 57 percent were readmitted within 4 years (Moos et al. 1994*a*,*b*). These rates are considerably higher than in comparable mixed-age or younger adult samples (Kastrup 1987; Woogh 1990).

Older and younger AOD-abuse patients received different types of treatment. Compared with younger patients, older patients received care focused more on their medical needs (e.g., detoxification) than on their AOD-abuse and psychiatric treatment needs (Moos et al. 1993). Moreover, despite the complexity and chronicity of their AOD-abuse problems, older patients were less likely to receive mental health aftercare (e.g., individual or group counseling following discharge) than were younger patients (Moos et al. 1995).

The readmission rates of older patients depended in part on their AOD-abuse histories. First-time AOD-abuse patients had lower readmission rates than the total sample (Moos et al. 1994*b*). This finding is consistent with the observation that late onset problem drinkers tend to have better prognoses compared with early onset problem drinkers (for reviews, see Atkinson 1995; Schonfeld and Dupree 1995).

Co-occurring psychiatric disorders also affected the frequency of health service use and complicated the treatment of older AOD abusers. At the index episode of care, almost 30 percent of the patients suffered from concomitant psychiatric disorders, most commonly depressive disorders, personality disorders, and schizophrenia (Moos et al. 1993). These dual-diagnosis patients used proportionally more health care resources. For example, during the 4 years after the index episode, more than 70 percent of dual-diagnosis patients were readmitted for inpatient treatment, compared with 57 percent of patients with only an AOD-abuse diagnosis.

In addition to AOD-abuse history and the presence of psychiatric disorders, the study identified several other predictors of higher readmission rates for older AOD-abuse patients at both the 1- and 4-year followups. These predictors included being unmarried, more prior service use, more severe and complex psychiatric diagnoses, disrupted or shorter length of treatment, and insufficient mental health aftercare (Moos et al. 1993, 1994*b*, 1995).

### Treatment Effectiveness

The question of treatment effectiveness among older AOD-abuse patients usually is framed as a comparison: Do older patients respond as well to AOD-abuse treatment as younger patients do? No definitive answer exists for this question (for reviews, see Atkinson 1995; Schonfeld and Dupree 1995). Clinical observation suggests that older AOD-abuse patients respond well to treatment approaches tailored to their needs, such as slower paced treatment sessions to accommodate cognitive decline and nonconfrontational counseling approaches. Some empirical evidence supports the idea that age-specific approaches increase treatment effectiveness (Atkinson 1995); however, these studies typically focus on programs that combine many treatment elements. It is therefore unclear which specific factors promote better outcomes for older clients.

Some recent investigations have begun to clarify this issue by examining the match between age and specific treatment modalities. For example, Rice and colleagues (1993) found that older adults did best in therapy with an individual focus, whereas younger patients did better in relationship-focused treatment. Analysis of the older VA patients for whom detailed treatment information was available demonstrated that these patients had better outcomes in programs with more structured program policies, flexible discharge rules, more comprehensive assessment, and extensive mental health aftercare. More intensive treatment was associated with poorer outcomes (Moos et al. 1995). In contrast, younger patients did better in programs that emphasized family involvement in assessment and treatment, community consultation, and the development of social and work skills (Moos et al. 1995). Family involvement and life-skills training may be less effective for older patients because their long-standing alcohol problems may have eroded their support from family, friends, and employers. Moreover, the kind of work- and social-skills development that is emphasized in the treatment of younger patients may not fit the developmental needs of older patients, who often are retired and have fewer family responsibilities.

## Future Directions

Although the studies reviewed here have contributed new data about late-life drinking behavior, many questions remain. For example, further information is needed on the relationships between life context and drinking behavior in later life. Moreover, longitudinal studies should investigate the predictors of late onset problem drinking and its remission. Researchers must identify factors that deter older adults from seeking assistance for their alcohol-related problems and devise new screening and referral methods that facilitate earlier recognition and treatment of late-life drinking problems.

The study of VA patients has demonstrated that older, treated AOD-abuse patients—especially those with long histories of alcohol abuse and psychiatric comorbidity—place a heavy burden on the health care system. Consequently, researchers and other professionals must work to evaluate and improve the treatment effectiveness in this patient population. Factors such as continuity of care and followup mental health care appear to promote better outcomes among older patients and may be especially important for patients who have fewer informal social resources from which to draw. Accordingly, researchers must determine how much and what kind of mental health aftercare is optimal for maintaining successful treatment outcomes among older patients.

Future research also should address the match between the treatment needs of older AOD-abuse patients and particular treatment program characteristics. Currently, the data more clearly indicate which treatment approaches do not work well for older patients than which ones do. More broadly, a need exists for further theory development in the study of late-life drinking behavior and for more longitudinal investigations of older adults’ drinking behavior, especially among understudied populations, such as older women and older adults in racial minority groups. Such work should enhance our understanding of the course and predictors of late-life drinking behavior and assist clinicians in treating older adults’ alcohol-related problems.

## Footnotes

1 As used in this article, the term “problem drinking” refers to any kind of alcohol consumption that results in physical, psychological, or social problems, whether or not it fulfills the diagnostic criteria of alcohol abuse or dependence.

2 The definitions of “older adults” widely vary in the research literature, ranging from people over age 50 to those over age 65. For this article, older adults are defined as people over age 55.

3 Participants were classified as “problem drinkers” if they had affirmative responses to the Drinking Problems Index (Finney et al. 1991), which assesses adverse physical, psychological, and social consequences of alcohol use.

4 To be classified as “in remission,” participants must have had no drinking problems according to the Drinking Problems Index.
